# Understanding Transcription Factors and How They Affect Processes in Cucumber Sex Determination

**DOI:** 10.3390/metabo13060740

**Published:** 2023-06-10

**Authors:** Szymon Turek, Aparna, Agnieszka Skarzyńska, Wojciech Pląder, Magdalena Pawełkowicz

**Affiliations:** Department of Plant Genetics, Breeding and Biotechnology, Institute of Biology, Warsaw University of Life Sciences, 02-776 Warsaw, Poland; szymon_turek@sggw.edu.pl (S.T.); aparna_aparna@sggw.edu.pl (A.); agnieszka_skarzynska@sggw.edu.pl (A.S.); wojciech_plader@sggw.edu.pl (W.P.)

**Keywords:** transcription factors, interactome network, sex development, sex determination, cucumber (*Cucumis sativus*)

## Abstract

Plant reproduction is a fundamental process on Earth from the perspective of biodiversity, biomass gain, and crop productivity. It is therefore important to understand the sex determination process, and many researchers are investigating the molecular basis of this phenomenon. However, information on the influence of transcription factors (TFs), genes that encode DNA-binding proteins, on this process is limited, although cucumber is a model plant in this regard. In the present study, based on RNA-seq data for differentially expressed genes (DEGs), we aimed to investigate the regulatory TFs that may influence the metabolic processes in the shoot apex containing the forming flower buds. Therefore, the annotation of the genome of the B10 cucumber line was supplemented with the assigned families of transcription factors. By performing ontology analyses of the DEGs, the processes they participate in were identified, and TFs were located among the results. In addition, TFs that have significantly overrepresented targets among DEGs were detected, and sex-specific interactome network maps were generated, indicating the regulatory TFs based on their effects on DEGs and furthermore, on the processes leading to the formation of different-sex flowers. Among the most overrepresented TF families in the sex comparisons were the NAC, bHLH, MYB, and bZIP families. An interaction network analysis indicated the most abundant families among DEGs’ regulatory TFs were MYB, AP2/ERF, NAC, and bZIP, and those with the most significant impact on developmental processes were identified, namely the AP/ERF family, followed by DOF, MYB, MADS, and others. Thus, the networks’ central nodes and key regulators were identified with respect to male, female, and hermaphrodite forms. Here, we proposed the first model of the regulatory network of TFs that influences the metabolism of sex development in cucumber. These findings may help us to understand the molecular genetics and functional mechanisms underlying sex determination processes.

## 1. Introduction

Cucumber (*Cucumis sativus*) is a globally significant vegetable crop and is also recognized as a model organism for exploring the intricacies of plant sex determination, encompassing male, female, and hermaphrodite forms. Although the process of sex determination in cucumbers is currently the focus of numerous scientific investigations, the underlying mechanisms of sex determination in this species remain incompletely understood.

The knowledge about the TFs that influence the metabolic processes involved in sex determination is also very limited. It will be very interesting to see which TFs and metabolic processes are involved in the process of sex determination in cucumber. With the increasing quantity of omics data such as sequenced genomes and transcriptomes, there is a strong basis and the opportunity to construct interactome networks that indicate the influence of regulatory TFs on selected processes.

Among the most well-known and described reference genomes of *Cucumis sativus* are: 9930, a Chinese line [1]; Gy14, a North American line [2]; and B10v3, a European line [3]. Recently, genomes were compared using structural data presented in databases as well as previously reported experimental data. It was shown that the B10v3 genome was the longest due to its 342.5 Mbp assembled length [4]. Among others, in order to obtain the most complete picture of the relevant elements, in this study, we focused on this version of the genome for further analyses. Understanding how genomes are organized is the basis for insight into the functioning of organisms. Knowledge of regulatory mechanisms and their links to metabolic processes is an important part of the interactions that control gene action.

TFs are proteins that control the activity of gene regulatory networks and cell type specification. They represent a class of essential regulatory proteins that are critical for controlling gene expression and modulating various physiological processes in plants, including their development, hormone signaling, and stress responses. TFs play an important role in the regulation of complex metabolic pathways in response to environmental and physiological signals. They are key regulators of plant primary and secondary metabolism, producing a large number of specialized metabolites with a wide range of functions and applications [5,6]. However, the inferred function of TF can be influenced by the genomic context in which it occurs. In addition, knowledge of the genome can be useful for the identification of novel TFs [7]. TFs bind to sequences of DNA, usually to motifs in the promoters of their target genes. Together with other proteins, such as transcription regulators (TRs), they regulate gene transcription [8]. The regulatory mechanism underlying gene expression mediated by TFs relies upon the fundamental process of binding to *cis*-regulatory elements located within gene promoters [9], influencing the expression of nearby genes. This regulatory mechanism plays an important role in orchestrating gene expression in plants [5].

In recent years, the identification and characterization of TFs have been made possible by the development of numerous databases, including iTAK [6], PlantRegMap [10], and CIS-BP [11], which encompass 197, 165, and 72 plant organisms, respectively. These databases contain detailed descriptions of broadly classified TF families for each species, thereby facilitating their efficient exploration. Furthermore, the iTAK database offers supplementary information on TRs that function by interacting with the basal transcriptional apparatus, which includes TFs [6]. This additional information is particularly valuable in expanding our understanding of the intricate mechanisms underlying gene expression regulation in plants. By leveraging the comprehensive information provided by these reference databases, researchers can efficiently search for TFs within the results of a transcriptome study experiment and examine their interactions with other components in the system being investigated. This provides a valuable framework for investigating the complex regulatory networks underlying various biological processes in plants, including sex determination in cucumber.

Our work can be divided into three main areas. The first part concerned the identification of TFs in the B10v3 cucumber genome by comparing annotated genes with other *Cucumis sativus* data available in databases. The second area of work was the functional analysis of differentially expressed genes (DEGs) associated with cucumber sex differentiation, which were detected in a previous study [12]. In order to extend the results of the functional analysis of the DEGs, a Gene Ontology (GO) analysis and a KEGG pathway analysis were carried out to identify the metabolic processes in which the DEGs are involved. Among the detected GO terms, enriched DEGs were localized and assigned as TFs. The third area of work concerned the search for DEGs’ regulatory TFs (described in the rest of the publication as regulatory TFs) presented as interactome networks. This step aimed to identify TFs that could potentially interact with DEGs and thus regulate their expression and the processes in which they participate. Analyzing DEGs and their involvement in biological processes, molecular functions, and metabolic pathways, as well as the detailed identification of regulatory TFs, was the aim of our work.

In this paper, we present the world’s first interactome map of regulatory TFs that influence DEGs encoding proteins involved in metabolic processes related to sex determination in cucumber and provide their functional characterization. We propose a multiomics approach to integrate the data and gain a complex view of the interplay between cell signaling and gene regulation in relation to plant sex determination.

## 2. Materials and Methods

**Figure 1:**
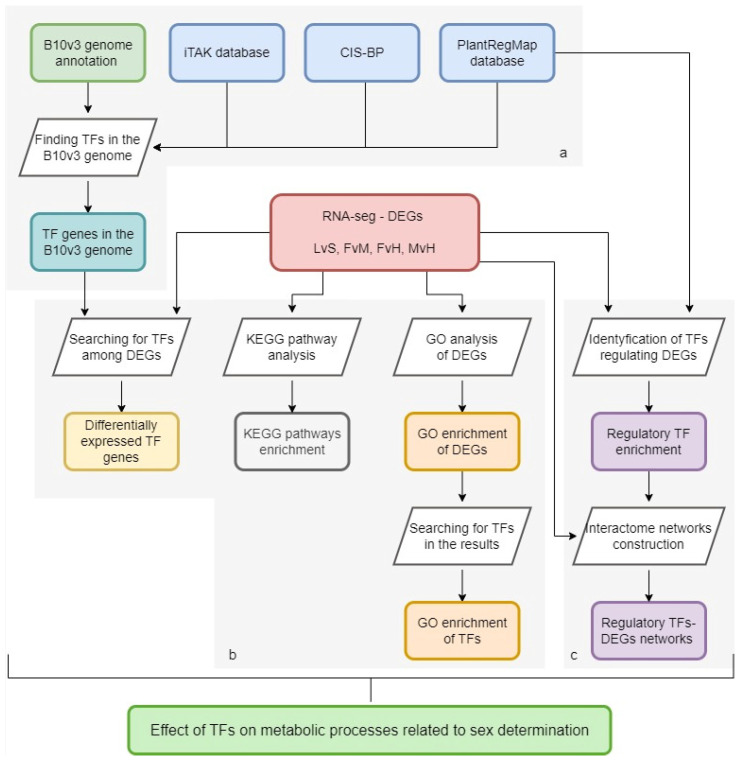
Diagram showing the analysis steps performed. Gray boxes indicate the main research areas: (**a**) a database of TFs in the B10v3 genome; (**b**) GO and KEGG enrichment in the pools of DEGs connected to sex determination and pointing out the TFs among them; (**c**) interactome network construction between regulatory TFs and DEGs.

A schematic representation of the analytical methodology employed for each of these discrete steps is provided in Figure 1, consisting of three areas: 1—a database of TFs in the B10v3 genome; 2—GO and KEGG enrichment in the pools of DEGs connected to sex determination [12] and pointing out the TFs among them; 3—interactome network construction between regulatory TFs and DEGs.

### 2.1. Finding the TFs in the B10v3 Genome

To identify TFs in the B10v3 genome, data from three databases were used: PlantRegMap, iTAK, and CIS-BP. The PlantRegMap and CIS-BP databases contain information on plant TFs, and PlantRegMap also provides available software to detect TFs in the genome. The iTAK database contains information on TFs, TRs, and protein kinases (PKs), as well as software for their detection in a given dataset. To detect the TFs in the B10v3 genome dataset, the longest amino acid sequences per gene were used as an input for TF identification. In order to work with the PlantRegMap database, it was necessary to obtain gene identifiers that were consistent with the identifiers contained in that database. This required the translation of the protein identifiers from the B10v3 genome to those in the database which were the Gy14v1 cucumber genome identifiers. For this purpose, the “ID mapping” tool that is available on the database server of PlantRegMap was used.

### 2.2. TFs among the Results of RNA-Seq Experiments

TFs were searched among DEGs (genes with a statistically significant differential expression from the RNA-seq experiment) from the shoot apex between cucumber lines differing in sex [12]. We analyzed DEGs in the leaves vs. shoot apex (LvS) and in the shoot apex based on the following three comparisons of flower sex types: female vs. male (FvM), female vs. hermaphrodite (FvH), and male vs. hermaphrodite (MvH). In the previous experiments, the sequencing reads were mapped to one of the first versions of the reference cucumber genome. Therefore, it was necessary to update information and connect them using the BLAST algorithm with the gene identifiers of the newest version of the genome—B10v3 [3]. Using the results of the RNA-seq experiment together with the information about TFs within the B10v3 genome (from the previous step in Section 2.1), DEGs were assigned to the TFs’ families, according to the PlantRegMap, CIS-BP, and iTAK databases.

### 2.3. Gene Ontology Analysis among DEGs

To gain further insight into the metabolic processes that might differentiate cucumber lines with different sexes, the ontology of the DEGs was examined using the GO Term Enrichment tool from the PlantRegMap database. This tool helps to identify significantly overrepresented GO terms or the parents of these terms in the selected gene set. Fisher’s exact tests were used to find significantly overrepresented GO terms, using a *p*-value threshold of 0.01. This analysis was based on the DEGs identified in the LvS, FvM, FvH, and MvH comparisons from the RNA-seq experiment [12].

### 2.4. KEGG Pathway Enrichment Analysis of DEGs

A KEGG pathway enrichment analysis was conducted for the DEGs identified from the LvS, FvM, FvH, and MvH comparisons. To use the KEGG database, gene identifiers from the *Cucumis sativus* 9930 genome were required. To obtain these identifiers, peptides encoded by DEGs were searched in the 9930 genome protein database using the BLASTP program. The ShinyGO server [13] was then used to perform the enrichment step of the KEGG pathways. The results were obtained using a standard FDR threshold value of 0.05. Finally, each of the genes that were part of the detected enriched KEGG terms was annotated according to the B10v3 genome annotation.

### 2.5. DEG’s Regulatory TFs Enrichment

The “TF enrichment” tool in the PlantRegMap database was used to determine the enrichment of regulatory TFs which interact with the DEGs. This tool makes it possible to find TFs that have significantly overrepresented targets in the gene group under study [10]. Transcriptional regulations for cucumber in the PlantRegMap database have been inferred by the combination of TF binding motifs and were used as background in our analysis. Fisher’s exact tests with a *p*-value threshold of 0.05 were performed to test whether there was a higher proportion of DEGs as TF targets. This method made it possible to identify regulations between TFs and DEGs and to also identify the number of TFs that had significantly overrepresented target number. Both regulatory TFs and targeted DEGs were used to build the interactome networks. They were prepared using the network D3 library in the R programming language [14]. This study determined the effect of the interaction relevance of the TF families on the DEGs based on the constructed networks.

## 3. Results and Discussions

### 3.1. TF Search Results in the B10v3 Genome

**Figure 2:**
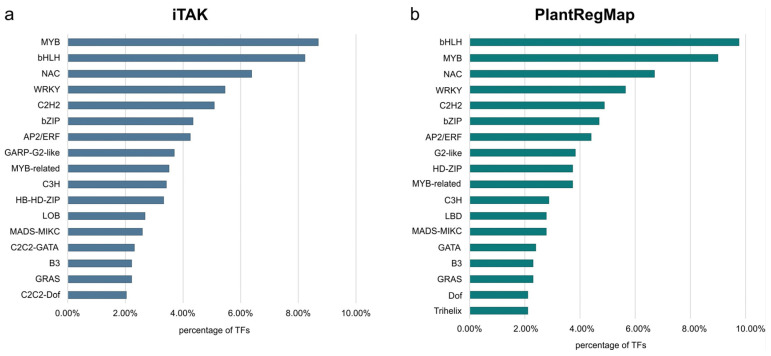
TF families detected in the B10v3 genome by the iTAK (**a**) and PlantRegMap (**b**) databases, which account for more than 2% of all identified TFs.

In order to obtain a complete view of the TFs across the genome, we carried out a whole genome analysis using B10v3 as a reference. Using the PlantRegMap and iTAK databases, it was possible to find TFs in the B10v3 cucumber reference genome. The results of the matching between the databases differed slightly, but for the most part, they remained consistent with each other. Based on the consensus rules for TF prediction and classification with the use of data from the PlantTFDB database, the iTAK database was created [6]. Annotations from the iTAK database were therefore used in the analyses. Using the “TF prediction” tool available in the PlantRegMap database, 1045TFs were assigned to the searched amino acid sequences. The same input file was used to search for TFs in the iTAK database, allowing for the annotation of 1082 TFs, 1355TRs and 656 PKs in the B10v3 genome. Assigning annotations from the CIS-BP database identified 973 TFs in the B10v3 genome. Of the 16,104 sequences in the B10v3 genome, 14,269 sequence identifiers were assigned from the PlantRegMap database (Supplementary Table S1). TF search results from the PlantRegMap, CIS-BP, and iTAK databases were added to the B10v3 genome annotation and supplemented with TR and PKs search results. The resulting B10v3 genome annotation is provided in Supplementary Table S2. For the results obtained using the PlantRegMap and iTAK databases, the percentage of detected TFs in genomes per family is shown in Figure 2. The prepared annotation of the B10v3 genome, supplemented with information on genes encoding TFs, RFs, and PKs, enriches the knowledge of the genome of the B10v3 cucumber line. The number of TF families detected in the cucumber genome corresponds to TFs detected in other plants [10]. The distribution of the identified genes is consistent with the factors detected in other plants, where the main TF families are MYB, bHLH, NAC, and WRKY [15].

### 3.2. TFs among DEGs

TFs have been identified among sex-specific DEG sets from RNA-seq analyses [12]. The results of the RNA-seq experiment supplemented with the annotations of TFs can be found in Supplementary Table S3. Among the DEGs from the tested comparisons, namely LvS, FvM, FvH, and MvH, 8.7%, 10.77%, 5.88%, and 10.91% of the DEGs were found to be TFs, respectively (Table 1).

**Figure 3:**
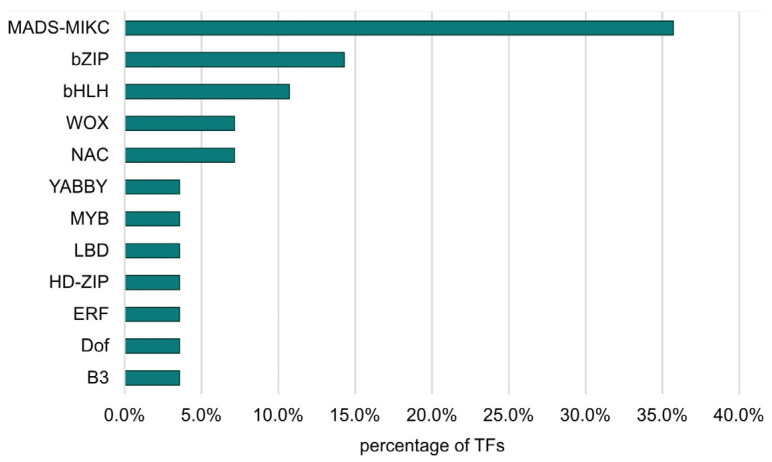
Percentage of TF families identified among all TFs that were DEGs in the FvM comparison.

**Figure 4:**
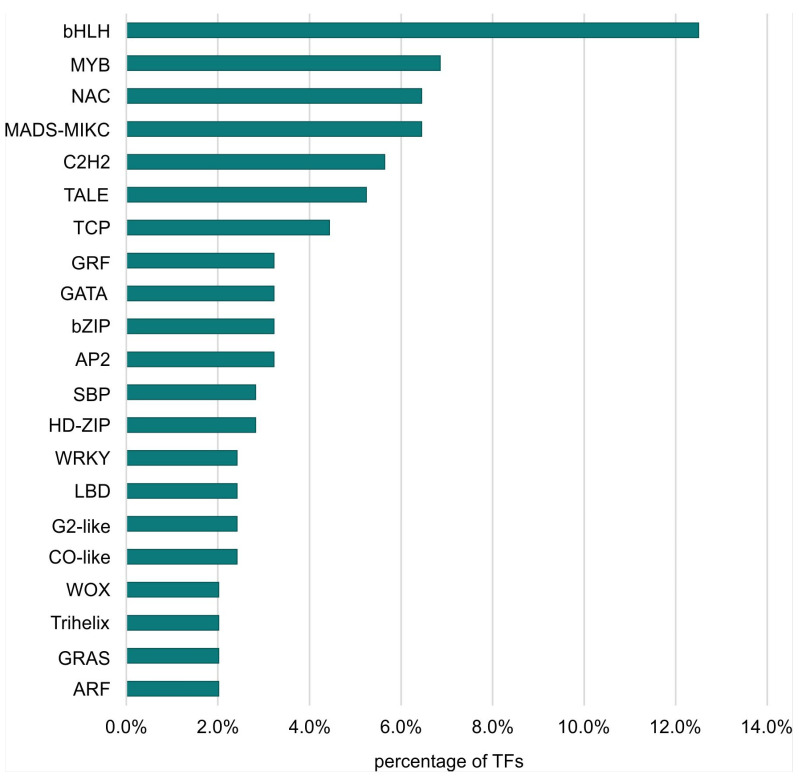
Percentage of TF families identified among all TFs that were DEGs in the LvS comparison.

The largest number of TFs was detected for the LvS comparison (8.70%). However, this number was due to the largest number of DEGs being detected between the leaf and shoot apex, containing genes responsible for the transition from the vegetative to the flowering phase. The DEG number in the shoot apex among male, female, and hermaphrodite lines was significantly lower, while the percentage of TFs detected for these comparisons remained similar. In the MvH comparison, where six TFs were detected, half of them belonged to the MADS-MIKC family. The other assigned families were HB-WOX, NAC, and C2C2-YABBY. For the FvH comparison, in which only two TF genes were detected, the bHLH and C3H families were defined. Figure 3 and Figure 4 show graphs with the highest number of TF genes detected in the FvM and LvS comparisons, respectively. For the FvM and MvH comparisons, the largest number of differentially expressed TFs belonged to the MADS-MIKC family. This represented a significant increase in the proportion of these TFs among DEGs (3.8% and 5.5%) relative to their contribution to the whole genome (0.1% of all genes in the genome are MADS-MIKC TFs). When comparing LvS, the TFs that were found in the highest abundance, i.e., bHLH, MYB, NAC, and C2H2, corresponded in abundance to the distribution of TFs across the genome. The TFs of the MADS-MIKC family in this comparison also represented an increased proportion relative to their presence in the reference genome (0.56%). MADS TFs are a family of DNA-binding proteins that play an essential role in various plant developmental processes, especially floral organ identity and differentiation [16,17], while additionally controlling the expression of genes that determine the identity and morphology of sepals, petals, stamens, and carpels. The MADS TFs in cucumber are similar to those in other plants, as they are also involved in the flowering time regulation and floral organ development [18]. The detection of the MADS-MIKC family as the most abundant among the TFs detected led us to link the MADS family to the ABC model of flower development [19].

In addition, the presence of eight differentially expressed TFs of the AP2 family is important to note, due to the link between sex determination processes and ethylene metabolism [20].

### 3.3. Ontology Analysis among DEGs

**Figure 5:**
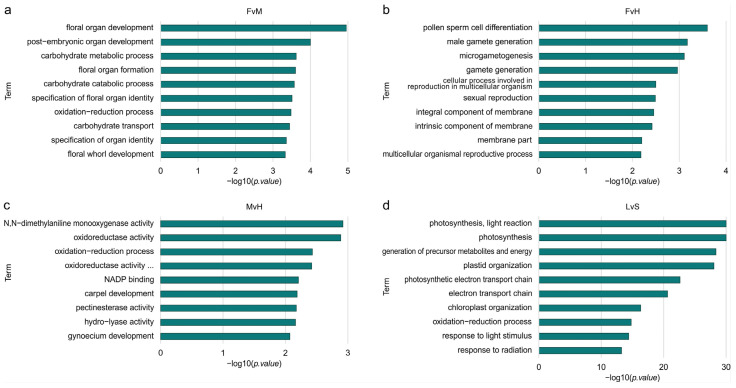
Box plots of significantly enriched GO terms for genes with significantly differential expressions for the FvM (**a**), FvH (**b**), MvH (**c**), LvS (**d**) comparisons.

The results of the GO term enrichment analysis, including the results of the statistical testing, can be found in Supplementary Table S4. For DEGs, an overrepresentation of GO terms was found in all comparisons—LvS, FvH, FvM, and MvH—as shown in Figure 5. For FvH, most of the GO terms were related to processes such as pollen sperm differentiation, male gametogenesis, or microgametogenesis differentiation, indicating significant relationships to processes involved in sex determination and male organ formation. In this comparison (FvH), female organs are formed in both female and hermaphrodite flowers, whereas male organs are only present in hermaphrodite flowers.

In the FvM comparison, the most significant enrichment concerned genes that are involved in the flower formation developmental processes, overall organ development, and carbohydrate metabolism processes.

Processes related to enzyme activity, such as monooxygenase, oxidoreductase, hydrolase, or pectinesterase, were the most enriched in the MvH comparison. Genes involved in the development of the carpels and gynoecium were also enriched. In this comparison, the female organs are formed in the hermaphrodite flowers, but in male flowers, the growth of this organ is inhibited, and thus the difference connected to these groups is expected. When comparing LvS, the enriched processes differed significantly from the other comparisons. The highest enrichment in that case was for processes related to photosynthesis, light response, and plastid or chloroplast organization. Comparing a vegetative organ such as a leaf with generative organs such as the whole structure of a shoot apex with small floral buds indicates which genes and processes differentiate these two organs. The number of DEGs was the highest in the LvS comparison and significantly exceeded the DEG number in the other comparisons. The analysis of the ontology network (Supplementary Figures S5–S8) showed significantly enriched processes, marked in red and yellow. For the FvH and MvH comparisons, the created ontology networks were significantly simpler than the other comparisons, namely the FvM and LvS comparisons, due to the smaller number of significantly DEGs identified. The FvH comparison represented the most enriched final processes, converging to a single final process of pollen germ cell differentiation. Similarly, for the MvH comparison, the structure of the ontology converged on final terms describing gynoecium development and carpel development, although a separate branch indicating metabolic processes and a final term describing oxidation and reduction processes were additionally described. In comparison, the FvH ontology network was much more extensive, where three main branches can be observed. The first corresponded to floral organ formation processes, the second indicated metabolic processes taking place, while the third described enriched processes related to transport. In the case of GO terms describing organ development, processes such as floral organ development and floral organ formation had the greatest enrichment. In addition, we could distinguish terms describing gynoecium development and carpel development. The processes responsible for oxidation–reduction and carbohydrate metabolism had the greatest enrichment among metabolic processes. The different branches of ontology converged to final terms describing processes related to the metabolism of pectin, salicylic acid, inositol, and fatty acids. A separate branch of the network described processes related to carbohydrates, saccharides, and sucrose transport.

**Figure 6:**
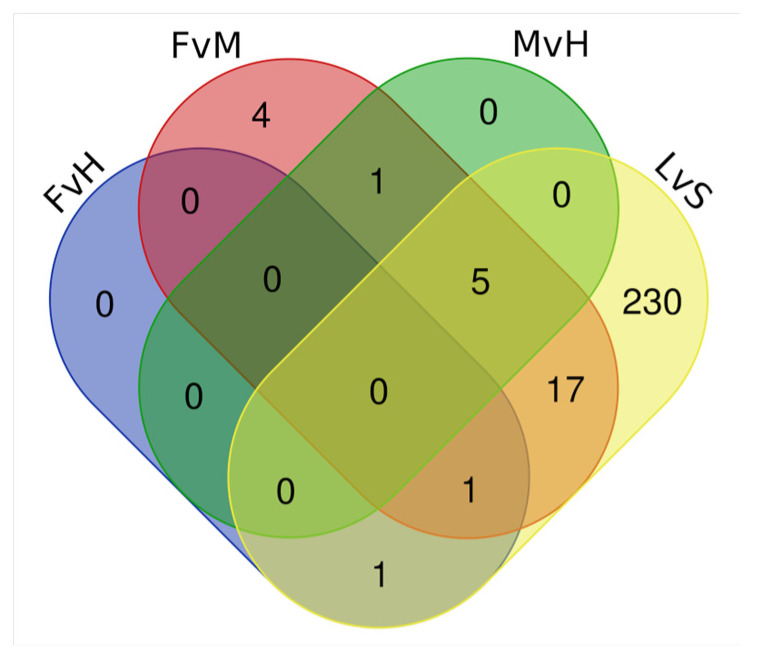
Venn diagram showing overlapping TFs among DEGs for the FvH, FvM, MvH, and LvS comparisons.

Subsequently, FvH, FvH, MvH, and LvS comparisons were carried out on the detected DEGs which were classified as TFs. The results of such comparisons are shown on a Venn diagram (Figure 6.). Among the detected TFs with differential expression, five of them were detected simultaneously in the FvM, MvH, and LvS comparisons. The genes encoding TFs belonging to the families were as follows: *Cucsat.PASA.G1363* (MADS-MIKC), *Cucsat.PASA.G2030* (WOX), *Cucsat.PASA.G9475* (YABBY), *Cucsat.PASA.G8448* (MADS-MIKC), and *Cucsat.PASA.G18435* (MADS-MIKC). A GO enrichment analysis of these genes, the results of which are included in Supplementary Table S9, identified the most enriched processes directly indicative of involvement in the sex development process: carpel development and gynoecium development (the *Cucsat.PASA.G9475* and *Cucsat.PASA.G8448* genes).

**Figure 7:**
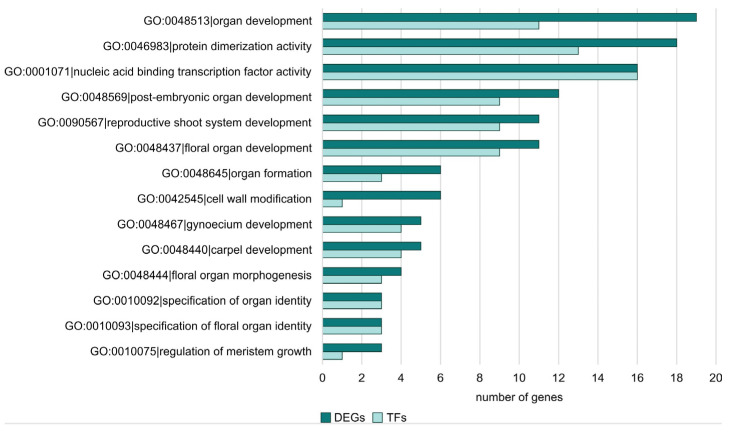
The number of TF genes among DEGs for FvM comparison with regard to enriched ontology terms.

The next step in the analysis was to check whether there were any DEGs in the enriched GO terms that were also TFs. For this purpose, we checked all DEGs in the sex-specific comparisons. In Figure 7, the frequency of TF genes among DEGs for each GO term is shown for the FvM comparison. It can be seen that TFs are responsible for floral developmental processes, carpel development, and organ formation. This indicates the actual involvement of TFs in processes linked to the plant’s sex development. No TFs were detected for the enriched GO terms in the FvH comparison. For the MvH comparison, two TFs involved in gynoecium and carpel development processes were detected among the enriched GO terms. They are also directly related to issues of plant sex development, which, as can be seen, demonstrates the relevance of TFs in this process. The LvS comparison was the most abundant. Therefore, it consisted of the largest number of TFs. The GO terms to which the TFs were assigned were related to metabolic processes and biosynthesis.

### 3.4. KEGG Enrichment Results in DEGs

**Figure 8:**
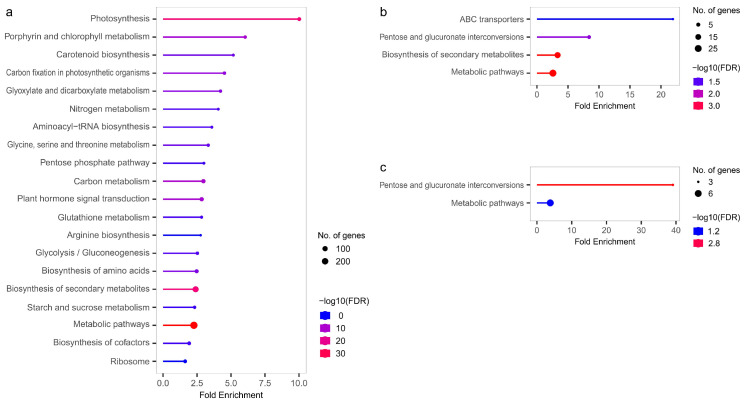
Graphs showing enriched KEGG pathways for LvS (**a**), FvM (**b**), and FvH (**c**) comparisons.

The enrichment analysis of KEGG pathways performed for DEGs (LvS, FvM, FvH, and MvH comparisons) allowed us to obtain a better understanding of the functions associated with DEGs and to perform the identification of metabolic processes involved in sex determination. The results of statistical testing of the KEGG pathway enrichment can be found in Supplementary Table S10. The KEGG enrichment results are shown in Figure 8. No statistically significant results were obtained for the FvH comparison. For the FvM comparison, the highest enrichment was related to the ABC transporters pathway, which refers to the ATP-binding cassette (ABC) transporters. For both the FvM and MvH comparisons, statistically significant enrichments were found for the pentose and glucuronate interconversion pathways. When comparing LvS, the most significant pathways included photosynthesis and metabolic processes. The annotated results of the KEGG analysis indicating which cucumber genes are part of a particular KEGG pathway are presented in Supplementary Table S11. No TFs were identified among the genes included in the enriched KEGG pathways, and only protein-coding genes were present. However, similar to the GO analysis, significant differences in overrepresented pathways could be observed between the generative (FvM and MvH comparisons) and vegetative (LvS comparison) organs.

### 3.5. DEGs’ Regulatory TFs

The study of flowering in cucumber is crucial due to its significant economic importance and vulnerability to both endogenous and exogenous factors. The impact of these factors collectively determines gene expression, which in turn is influenced by the activity of various TFs. The regulatory TFs were assigned to families and functionally curated.

Our study showed regulatory TF interactions and their influence on DEGs; thus, taking these results together, we can answer the question as to which TFs influence flower morphogenesis at the early growth stages.

#### 3.5.1. DEG’s Regulatory TFs’ Enrichment Results

The results of the enrichment step carried out by performing Fisher’s exact tests made it possible to find TFs with significantly overrepresented target genes in the set of genes that were DEGs for the FvM, FvH, MvH, and LvS comparisons. The results obtained are included in Supplementary Table S12, together with additional information on the TF families derived from the created annotation for the B10v3 genome. A summary of the number of enriched TFs that had significantly overrepresented target number for the comparisons studied is shown in Table 2 below.

The analysis of the detected TF families showed that many TFs from the NAC family had a significantly enriched number of targets for the FvH and MvH comparisons. In addition, genes encoding TF families, such as bHLH and MYB, showed an increased number among the enrichment results for each comparison. For the LvS comparison, the highest number of TFs among the enrichment results belonged to the bZIP family, indicating differences between that comparison and the other comparisons. Using Fisher’s test, it was detected in each comparison whether there was a greater proportion of genes that were targets of a TF, compared with all the interactions described for cucumber in the PlantRegMap database. The individual genes detected could be considered as genes encoding TFs that could potentially affect the regulation of DEGs in this case. Although some of the families were significantly more frequent in the enrichment results, each of the genes encoding TFs in these results had a statistically significant overrepresented target DEG. The results obtained from such tests could also be considered in relation to the whole network of interactions between TFs and DEGs, which was also prepared in this study.

#### 3.5.2. Interaction Networks between Regulatory TFs and DEGs

**Figure 9:**
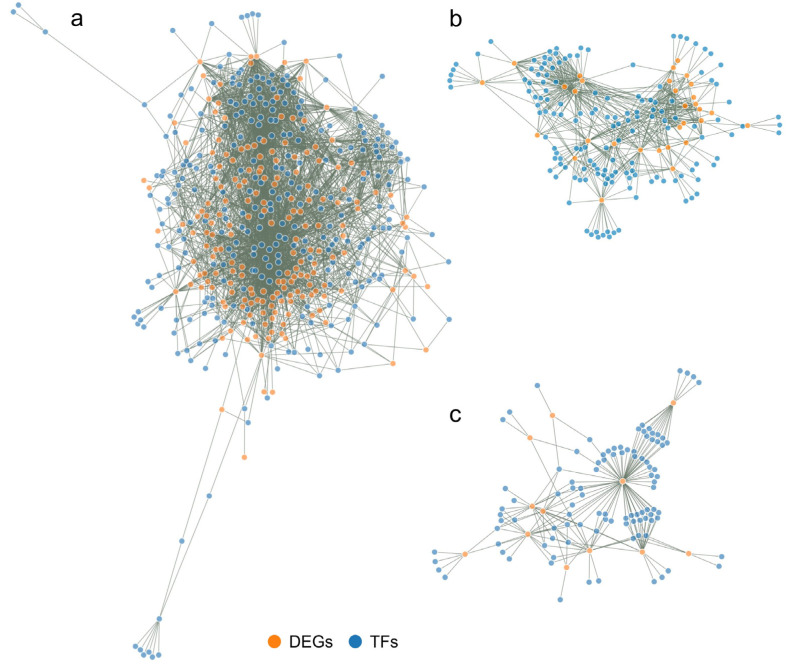
Static image of interactions networks between TFs and DEG targets in the FvM comparison (**a**), FvH comparison (**b**), and MvH comparison (**c**). Orange indicates DEGs, while blue indicates regulatory TFs.

The created interaction maps between regulatory TFs and their target DEGs are highly complex and is therefore presented in the form of interactive networks available in HTML files (Supplementary Files S13–S16). The advantage of such a network presentation is that it is possible to view the gene of interest together with the genes with which it interacts (DEGs or regulatory TFs), and to read their annotation. A static image of the networks for the FvM, FvH, and MvH comparisons is presented in Figure 9.

In the FvM comparison, the number of DEGs was significantly higher than that for the FvH and MvH comparisons. The FvM interaction network thus contained many more connections, showing that the male and female lines had significant differences in expressed genes. The FvH and MvH lines were thus simplified models, as the number of DEGs was much smaller but referred to processes associated with sex variation.

The resulting interaction networks between regulatory TFs and DEGs varied in complexity, depending on the number of DEGs used to construct the network. The largest network was created for the LvS comparison and consisted of 3029 nodes. The network for the FvM comparison consisted of 468 nodes, that for the FvH comparison consisted of 177 nodes, while that for the MvH comparison consisted of 191 nodes. The number of individual regulatory TFs and DEGs used to construct the networks is shown in Table 3. The smallest networks were created for the FvH and MvH comparisons, due to the flowers possessing a common element in the flower architecture. The FvM network was more developed due to the flower architecture concerning the distinction of the generative organ. 

**Figure 10:**
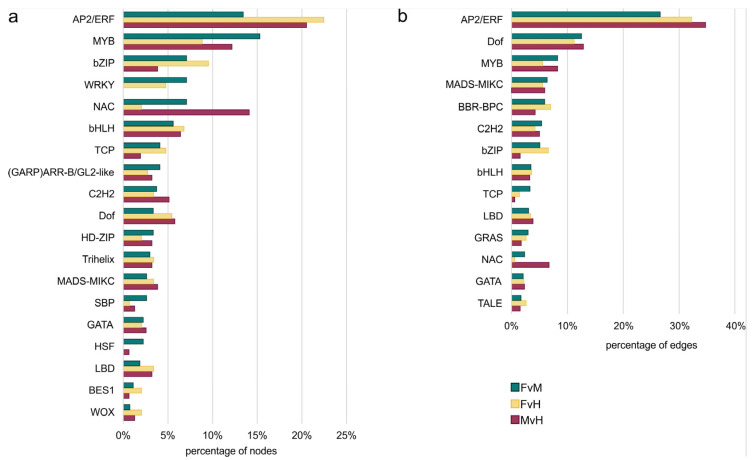
The percentage of nodes represents TFs from different family (**a**) and the percentage of edges to TF family (**b**) among the different comparisons.

In order to accurately characterize the network, we grouped up the TFs that influenced DEGs into 34 families and performed a functional characterization (Supplementary Table S17). This allowed us to elucidate which and how the regulatory TFs influenced DEGs and thus the proteins involved in metabolic processes in lines varying in sex in cucumber. In order to check the degree of the influence of regulatory TF families, the links between TFs and their targets were counted (Figure 10).

TFs are proteins that help to “turn on” or “turn off” certain genes by binding to the promoter, thereby regulating the functioning of the organism. An analysis of the interaction network identified several TF families that greatly influenced the expression of DEGs in male, female, and hermaphrodite flowers. The most numerous families of regulatory TFs in all three networks were the AP2/ERF (101), MYB (73), NAC (44), bZIP (39), and bHLH (35) families. Other families were less numerous in the sum of the three comparisons (Figure 9). However, some TFs were specified only for one comparison, namely FvM—YABBY, LFY, SRS, and EIL—or only for two comparisons, such as FvH and FvM—WRKY, CPP, and FAR1—and FvM and MvH—the HSF family. In terms of edge numbers, that is, family interactivity, which can be translated into the power to influence DEGs, the most numerous families in the three networks were also AP2/ERF (1069), DOF (465), MYB (296), MADS-MIKC (233), and BBR-BBC (219). Other families possessed fewer than 200 connections. Table 4 presents the top 10 TFs that had the most connections in each interaction network. 

Having the results on the enrichment of TFs possessing significantly overrepresented targets as DEGs and the TF–DEG interaction networks established, the question arises as to how the identified TF families influence sex determination, in which processes they are involved, and with which interaction. The hormonal regulation plays a crucial role in the process of sex determination, as the genes primarily involved in this process are associated with ethylene synthesis, such as *CsACS1*, *CsACS2*, and *CsACS11,* and they are linked with the genetic loci *F*, *M*, and *A*, respectively [21,22,23,24]. The expression and interactions among all three genes help in the development of female flowers in cucumber. TFs act as a very important factor that can either activate or repress the gene expression. Ethylene is the principal hormone that is responsible for the formation of specific organs and genes responsible for ethylene biosynthesis that have a direct association with the development of female flowers in cucumber [25,26]. In species such as *Zea mays* and *Cucumis melo*, ethylene leads to female flower formation, while it has the opposite role in *Citrullus lanatus* [21]. The ethylene production in the cucumber shoot apex primordia can readily modify the male to female flower ratio on the plant. It is known that a cucumber plant’s sex is linked to hormonal regulation, and ethylene plays an important role in this. Of the identified TF families, ten are associated with the ethylene response in other plants, and these are: AP2/ERF [27], MYB [28], NAC [29], bZIP [30], bHLH [31], WRKY [32], TCP [33], C2H2 [34], TALE [35], MADS-MIKC [36]. Additionally, other hormones such as auxin and cytokinin exert a positive effect on female sex determination through interactions with ethylene biosynthesis and signaling pathways [37,38]. The families connected with other hormones were also identified in this study: for auxins, NAC [39], bHLH [40], and LFY [41]; for cytokinin, bHLH [40] and BBR-BPC [42,43]. The results of other studies demonstrated that gibberellins (GA) can have dual effects on cucumber sex expression, inhibiting femaleness and inducing maleness, and an expression analysis has shown that *CsACS1G* transcription is promoted by auxins and inhibited by gibberellic acid [44,45]. According to the literature, the LFY [46], YABBY [47,48], MYB [49], BBR-BPC [42,43], and TALE [50] families are correlated with this hormone. AP2/ERF family members were identified on TF–DEG interaction networks as one of the largest groups of TFs in this study in all three comparisons (FvM, FvH, MvH). AP2/ERF family members induce ethylene signaling and flowering [27,51,52]. *CsACS11* is one of the ethylene biosynthesis genes [53] and is also thought to be a sex gene (*a*) in cucumber [22]. So far, it is not clear how the hormonal signaling pathways influence sex at the molecular level, so a further detailed analysis of the link between the regulatory TFs is needed. The occurrence of TFs belonging to the EILs family is also interesting because, according to the literature, EIL genes are key members of the ethylene signaling pathway, which is thought to play a crucial role in forming female flowers in cucumber. Ethylene not only controls female flower development but also suppresses development through the EIN2-EIN3/EIL1 signaling pathway. Cosegregation of the ETHYLENE-INSENSITIVE3 (EIN3)-like gene with the *M* locus has been observed in *Cucumis sativus*, suggesting its involvement in the sex determination process [54].

The formation of a complex flower architecture involves the MADS family described above. For the identified families, we found numerous links between TFs and DEGs and flower development. Several studies reported the role of the MADS-MIKC [36,55,56], bHLH [57,58], bZIP [59], and NAC [29,60,61] families in promoting or delaying flower development. The TALE family shows interactions with ethylene and cytokinin signaling [35]. Together with floral development, the timing of flowering is also crucial in plants for fruit and seed production. The MYB [62], bZIP [63], bHLH [57,58], and WRKY [64] families are involved in flowering time regulation, as described previously. A study in tomato showed that bHLH acts with SFT or LFY and controls flowering time. It also influences ethylene biosynthesis genes, as the expression of ethylene biosynthesis genes is upregulated in the overexpression line of bHLH [65]. In the forming gynoecium of the *Arabidopsis* flower, other hormones such as auxin and cytokinin interact with bHLH [40]. BBR-BPC/GAGA has been described in *Arabidopsis* to regulate the phytohormonal signaling of cytokinins, brassinosteroids, and ethylene [42,43]. The DOF TF has been reported to be involved in tissue differentiation, cell expansion, seed development, anther or pollen development, and flowering in plants [66,67,68]. The DOF family has also been implicated in the formation of vascular tissue in reproductive organs [69]. The interaction between the bZIP member and the C2H2 member in melon inhibits the development of the carpel in male flowers [70,71]. It also represses the transcription of ethylene biosynthesis genes [72]. The present study of TF–DEG interactions revealed that the TFs of the WRKY family were present solely in the FvH and FvM comparisons, while being absent in the MvH comparison. The WRKY family’s TFs interact with various flowering genes to regulate flowering timing in plants [64,73]. Another TF, YABBY, was exclusively found in the FvM comparison. The YABBY family’s TFs were described as playing a crucial role in anther and pollen sac development in cucumber, *Arabidopsis*, and rice [74,75,76,77]. The TFs from the YABBY family interact with MADS-box to control its expression during carpel development [78]. In addition, only the LFY TF was identified in the FvM comparison, which is known to respond to auxin and regulate flowering initiation, as presented in *Arabidopsis* [41,79].

Our analyses demonstrated the link between regulatory TFs and various developmental processes, including flower morphogenesis, flowering timing, and interactions with phytohormones. Gene expression governing specific functions involves the action of TFs. By conducting a detailed examination of interaction networks, we identified regulatory TFs that have the potential to regulate a significant number of DEGs. These regulatory TFs act as central hubs in the network and can influence a large portion of the nodes, thereby characterizing them as master regulators/hot links. The identification of these master regulators can serve as a valuable hub point for future investigations. By selectively focusing on these factors, their regulation or knockout may be utilized to observe changes in DEGs within the context of sex comparison in cucumbers. This approach holds considerable potential for expanding our understanding of the complex regulatory networks underlying sex development in cucumber.

In conclusion, in order to study the process of sex determination in cucumber, it is necessary to know how the genes responsible for this process are regulated. One potential mechanism that can alter gene expression is transcription-factor-mediated regulation. In our analysis, we used ontology analyses to look for processes that clearly indicated a link between DEGs (as well as TFs that were DEGs) and the cucumber sex determination process. These processes included, for example, the development of the carpels and the gynoecium. Having obtained a list of DEGs involved in the cucumber sex determination process, we sought to establish how they were regulated by TF. The establishment of an interaction network between DEGs and TFs made it possible to show the complexity of this process and, by analyzing the network, it was possible to identify numerous families of TFs that were linked to sex determination processes such as AP2/ERF, MYB, NAC, and MADS-MIKC. Using an enrichment analysis, it was also possible to detect genes encoding TFs that had significantly overrepresented targets as DEGs. The most abundantly enriched genes encoding TFs included the NAC, bHLH, MYB, and bZIP TF families. The KEGG analysis for DEGs indicated biochemical pathways and thus made it possible to identify which metabolic pathways were affected by TFs in connection with sex development. The work conducted in the analysis allowed us to illustrate the complexity of the interaction of TFs with DEGs, showing a potentially useful model for the mechanism of the regulation of the processes responsible for cucumber sex determination by TFs.

## Figures and Tables

**Table 1:** Number and percentage of TFs found in the list of genes with significantly differential expression for LvS, FvM, FvH, and MvH comparisons.

Comparison	Number of DEGs	Number of TFs Found for the Comparison	% of all DEGs
LvS	2852	248	8.70%
FvM	260	28	10.77%
FvH	36	2	5.88%
MvH	55	6	10.91%

**Table 2:** Table presenting the assignment of TFs having a significantly enriched number of target DEGs to TF families.

TF Family	Count for FvH	Count for FvM	Count for MvH	Count for LvS
bHLH	12	3	3	0
NAC	9	0	14	2
bZIP	6	0	0	0
E2F/DP	3	0	0	0
BBR-BPC	3	0	0	3
FAR1	2	0	0	0
MYB	2	5	2	10
ERF	2	0	0	0
ARR-B	1	1	1	0
MADS-MIKC	1	0	0	1
BES1	1	0	0	0
AP2	1	0	1	1
GATA	1	0	0	0
C2H2	1	0	4	5
CPP	1	0	0	0
Dof	1	1	3	4
WOX	1	0	1	0
HD-ZIP	0	2	6	2
G2-like	0	1	0	1
SBP	0	2	0	0
TCP	0	1	0	7
ARF	0	1	0	0
Trihelix	0	1	0	0
C3H	0	0	2	0
WRKY	0	0	1	6
MYB-related	0	0	0	0
GRAS	0	0	0	1
TALE	0	0	0	1
EIL	0	0	0	4

**Table 3:** The number of DEGs and regulatory TFs that were used to create the interactome network for the FvM, FvH, MvH, and LvS comparisons.

	FvM	FvH	MvH	LvS
DEGs	186	31	35	244
TFs	282	147	156	2785

**Table 4:** Top 10 number of TFs from established interaction networks that had the most connections to DEGs.

Comparison	Transcription Factor			No. of Edges
	Gene ID	TF Family	Description	
FvM	*Cucsa.362960*	MADS-MIKC	MADS box transcription factor	116
	*Cucsa.277740*	AP2	AP2-like ethylene-responsive transcription factor	109
	*Cucsa.026600*	BBR-BPC	GAGA-binding transcriptional activator	93
	*Cucsa.307870*	C2H2	Transcription factor IIIA	82
	*Cucsa.102120*	Dof	Dof zinc finger protein	81
	*Cucsa.280310*	GRAS	DELLA protein GAI	81
	*Cucsa.213830*	Dof	Dof zinc finger protein	76
	*Cucsa.159750*	BBR-BPC	GAGA-binding transcriptional activator	69
	*Cucsa.341290*	Dof	Dof zinc finger protein	60
	*Cucsa.098430*	Dof	Dof zinc finger protein	48
FvH	*Cucsa.362960*	MADS-MIKC	MADS box transcription factor	23
	*Cucsa.026600*	BBR-BPC	GAGA-binding transcriptional activator	21
	*Cucsa.277740*	AP2	AP2-like ethylene-responsive transcription factor	19
	*Cucsa.307870*	C2H2	Transcription factor IIIA	17
	*Cucsa.159750*	BBR-BPC	GAGA-binding transcriptional activator	14
	*Cucsa.280310*	GRAS	DELLA protein GAI	13
	*Cucsa.353140*	TALE	Homeobox protein knotted-1-like 1	13
	*Cucsa.102120*	Dof	Dof zinc finger protein	12
	*Cucsa.213830*	Dof	Dof zinc finger protein	12
	*Cucsa.098430*	Dof	Dof zinc finger protein	9
MvH	*Cucsa.362960*	MADS-MIKC	MADS box transcription factor	21
	*Cucsa.102120*	Dof	Dof zinc finger protein	17
	*Cucsa.277740*	AP2	AP2-like ethylene-responsive transcription factor	16
	*Cucsa.213830*	Dof	Dof zinc finger protein	12
	*Cucsa.307870*	C2H2	Transcription factor IIIA	12
	*Cucsa.341290*	Dof	Dof zinc finger protein	12
	*Cucsa.026600*	BBR-BPC	GAGA-binding transcriptional activator	11
	*Cucsa.159750*	BBR-BPC	GAGA-binding transcriptional activator	11
	*Cucsa.136780*	ERF	Dehydration responsive element binding transcription factor	9
	*Cucsa.237150*	ERF	Ethylene-responsive transcription factor ERF021	9
LvS	*Cucsa.362960*	MADS-MIKC	MADS box transcription factor	1579
	*Cucsa.277740*	AP2	AP2-like ethylene-responsive transcription factor	1485
	*Cucsa.026600*	BBR-BPC	GAGA-binding transcriptional activator	1324
	*Cucsa.307870*	C2H2	Transcription factor IIIA	1205
	*Cucsa.280310*	GRAS	DELLA protein GAI	1090
	*Cucsa.102120*	Dof	Dof zinc finger protein	1076
	*Cucsa.213830*	Dof	Dof zinc finger protein	1031
	*Cucsa.159750*	BBR-BPC	GAGA-binding transcriptional activator	1000
	*Cucsa.353140*	TALE	Homeobox protein knotted-1-like 1	920
	*Cucsa.341290*	Dof	Dof zinc finger protein	823

## Data Availability

Publicly available datasets were analyzed in this study, according to cited articles.

## Supplementary Materials

The following supporting information can be downloaded at: https://www.mdpi.com/article/10.3390/metabo13060740/s1. Table S1: S1.PlantRegMap_ID_mapping.txt; Table S2: S2.B10v3_annotation.csv; Table S3: S3.RNA_seq_results_annotated.xlsx, Table S4: S4.GO_enrichment_results.xlsx; Figure S5: S5.FvM_GO_enrichment_BP.png; Figure S6: S6.FvH_GO_enrichment_BP.png; Figure S7: S7.MvH_GO_enrichment_BP.png; Figure S8: S8.LvS_GO_enrichment_BP.png; Table S9: S9.Venn_set_enrichment_result.txt; Table S10: S10.KEGG_enrichment_results.xlsx; Table S11: S11.annotated_KEGG_results.xlsx; Table S12: S12.TF_enrichment.xlsx; File S13: S13.FvM_graph.html; File S14: S14.FvH_graph.html; File S15: S15.MvH_graph.html; File S16: S16.LvS_graph.html; Table S17: S17.annotated_networks_data.xlsx.
